# HEALTH CARE COST BURDEN AND SELF-REPORTED FREQUENCY OF DEPRESSIVE/ANXIOUS FEELINGS IN OLDER ADULTS

**DOI:** 10.1093/geroni/igae098.2453

**Published:** 2024-12-31

**Authors:** Namkee Choi

**Affiliations:** The University of Texas Austin, Austin, Texas, United States

## Abstract

Many older adults are burdened by out-of-pocket healthcare costs. Using the 2018-2021 National Health Interview Survey data, we examined: (1) healthcare cost burden and its associations with depressive/anxious feelings; and (2) whether during the pandemic (2020-2021), COVID infection and COVID-related healthcare access problems would also be associated with a higher likelihood of reporting healthcare cost burden and depression/anxiety. Nearly 12% of older adults (age 65+) reported healthcare cost burden, and a little over 18% reported daily/weekly depressive/anxious feelings. In generalized linear modeling analyses, the risk of healthcare cost burden was higher among women, racial/ethnic minorities (Blacks: incident risk ratio or IRR=1.49, 95% CI=1.35-1.65; Hispanics: IRR=1.27, 95% CI=1.12-1.44), those with chronic illnesses, mobility impairment, and those with Medicare Part D (IRR=1.14, 95% CI=1.04-1.20), but lower among individuals with Medicare-Medicaid dual eligibility, Medicare Advantage, VA/military insurance, and private insurance. The risk of daily/weekly depressive/anxious feelings was higher among healthcare cost burden reporters (IRR=1.76, 95% CI=1.64-1.88), controlling for sociodemographic and health status characteristics. The COVID-19 pandemic-related delay of or lack of access to medical care was also associated with a higher risk of reporting healthcare cost burden (IRR=1.67, 95% CI=1.52-1.84) and depression/anxiety (IRR=1.40, 95% CI=1.29-1.52). In sum, healthcare cost burden reporters were 1.8 times more likely to report high-frequency depressive/anxious feelings. Healthcare and social service providers should recognize the interconnection between financial strain associated with physical health problems and mental health in older adults. Policies and programs are needed to alleviate healthcare cost burden and ensure adequate access to healthcare services.

